# Evolution of the Numerical Model Describing the Distribution of Non-Metallic Inclusions in the Tundish

**DOI:** 10.3390/ma14092229

**Published:** 2021-04-26

**Authors:** Tomasz Merder, Jacek Pieprzyca, Marek Warzecha, Piotr Warzecha, Artur Hutny

**Affiliations:** 1 Faculty of Materials Engineering, Silesian University of Technology, Krasinskiego 8, 40-019 Katowice, Poland; jacek.pieprzyca@polsl.pl; 2 Faculty of Production Engineering and Materials Technology, Czestochowa University of Technology, al. Armii Krajowej 19, 42-201 Czestochowa, Poland; marek.warzecha@pcz.pl (M.W.); piotr.warzecha@gmail.com (P.W.); artur.hutny@pcz.pl (A.H.)

**Keywords:** continuous casting, tundish, nonmetallic inclusions, numerical modeling, physical modeling

## Abstract

Continuous casting is one of the steel production stages, during which the improvement in the metallurgical purity of steel can be additionally affected by removing nonmetallic inclusions (NMIs). This can be achieved by means of various types of flow controllers, installed in the working space of the tundish. The change in the steel flow structure, caused by those flow controllers, should lead to an intensification of NMIs removal from the liquid metal to the slag. Therefore, it is crucial to understand the behavior of nonmetallic inclusions during the flow of liquid steel through the tundish, and particularly during their distribution. The presented paper reports the results of the modeling studies of NMI distribution in liquid steel, flowing through the tundish. CFD modeling methods—using different models and computation variants—were employed in the study. The obtained CFD results were compared with the results of laboratory tests (using a tundish water model). The results of the performed investigations allow us to compare both methods of modeling; the investigated phenomena were microparticle distribution and mass microparticle concentration in the model fluid. The validation of the CFD results verified the analyzed computation variants. The aim of the research was to determine which numerical model is the best for describing the studied phenomenon. This will be used as the first phase of a larger research program which will provide for a comprehensive study of the distribution of NMIs flowing through tundish steel.

## 1. Introduction

The continuous steel casting method is currently one of the most advanced metallurgical technologies, which, nevertheless, is still being developed and upgraded. The aim of those activities, in addition to assuring the safety of the process, is to enhance the quality of the continuous castings process. A criterion commonly used for the quality assessment of continuous castings is the nature of their primary structure and their metallurgical purity defined mainly by the quantity, size, and distribution of nonmetallic inclusions (NMIs). The tundish is one of the final elements of the technological line, in which appropriate conditions prevail for refining steel to clear it of inclusions. Therefore, aside from research on refining in the ladle, investigations are conducted to identify the hydrodynamic conditions prevailing in the tundish [1,2,3,4,5,6,7,8,9,10,11], and also to determine the behavior of NMIs in the tundish [12,13,14,15,16,17,18,19,20,21,22,23,24,25,26].

In physical modeling testing, the main problem in the identification of the motion of non-metallic inclusions in the liquid steel flowing through the tundish is to define the conditions of similarity of interaction between steel flow velocity and the non-metallic inclusion flotation velocity. The main equations used for describing the flow of liquid steel and are the Navier–Stokes equations and the Newtonian stream continuity equations. Meanwhile, for determining the similarity of particle motion, Stoke’s law and the Froud (Fr), Reynolds (Re), and Archimedes (Ar) criterial numbers are used. This enables the selection of the type of NMI-simulating microparticles [25,26,27] to be made appropriately for given testing conditions. The investigations reported in studies [13,14,15,17,19,21,22,23,24] chiefly concern the issues of removing NMIs and the analysis of this phenomenon. Tests using water models, reported in the literature of recent years, have deal primarily with achieving a high level of microparticle (NMI) removal by blowing inert gas into the shield tubes [17], using shield tubes [19], gas curtains [13,14,15], tunnel filters [20], or using the phenomenon of microparticle agglomeration due to mutual collision [16].

In the area of numerical studies, the problem of liquid steel NMI removal is limited to utilizing the capabilities implemented in the codes of commercial mathematical models. The motion of inclusions, and more precisely the trace of their trajectory, can be determined using the Lagrange method [28], or the predefined equation of NMI transport conveyed in the vector fluid velocity field, considering forces determined by flow turbulence.

NMIs in liquid steel, modeled in CFD computations, are idealized spherical forms defined by their dimensions and densities. The real range of nonmetallic inclusion density variation assumed in computation [12,13,18,25,29,30,31] results from the fact that the aluminum oxide inclusions form clusters of an expanded structure and a variable Al_2_O_3_ fraction relative to the bonding metallic phase. In the majority of those studies, the boundary conditions reflect the entrance of NMIs (and their quantity and size) to the tundish with liquid steel through the surface corresponding to the inlet.

In the investigations reported in references [24,29,30,31,32], it is noteworthy that they use standard boundary conditions that do not match the real conditions. The boundary conditions for simulating the processes of NMI removal at the interfaces (liquid steel–liquid slag or liquid steel–refractory lining), available in commercial computation codes, are insufficient. In many numerical solutions, it is assumed that with the collision of NMIs with the liquid steel–tundish lining interface, the particle rebounds elastically from the lining wall [25,30]. This precludes a possible adhesion of NMIs to the lining and a permanent bond with the tundish lining material. There are also investigation results in the literature, in which it is assumed that NMIs in the metal bath will necessarily be absorbed at their first contact with the tundish lining [12].

A considerable number of CFD computation reports are also done with the assumption that all NMIs are captured by the slag once they reach the liquid metal–slag interface [24,29,30,31,32]. Therefore, the assumption is made in computations that the perfect absorption takes place. Studies were also conducted, such as [18,33,34,35], where the standard boundary condition at the interface under consideration is modified by using their own UDF functions. This modified condition concerned either the rebound or absorption of a given particle, depending on the critical velocity attained by that particle [18,34].

Another fact that is also worth of noting is that the main subject of those studies was the issue of removing NMIs from the liquid steel. The literature on the subject very rarely reports studies on the distribution of NMIs in liquid steel, which has a significant influence on the course of the refining process [34,35].

The presented paper reports the results of the modeling studies of NMI distribution in liquid steel, flowing through the tundish. CFD modeling methods—using different models and computation variants—were employed in the study. Numerical investigations were carried out for four simulation variants. Various models describing two-phase flow, namely the DPM (Discrete Phase Model), the Euler–Euler model, and the VOF (Volume of Fluid) model, as well as different turbulence description models, i.e., the RANS (Reynolds–Averaged Navier–Stokes) and the LES (Large Eddy Simulation), were employed. The numerical analysis covered four microparticles of a size about 20, 50, 100, and 140 μm, respectively. Nevertheless, numerical models need to be verified experimentally. This was done by measurements made on a water (physical) model of the plant. The laboratory tests were carried out on a water model using hollow glass microspheres that represented NMIs. The Abakus mobile fluid laser particle counter was used in the experiments. The obtained CFD results were compared with the laboratory test results. The comparison was made through qualitative and quantitative analyses. The qualitative analysis was based on the comparison of the motion of microparticles visualization, while the quantitative analysis, on the measurements of the concentration of microparticles in liquid. The validation of the CDF results verified the analyzed computation variants. It was shown that the flow and distribution of particles are best represented by the DPM_RANS numerical model. The developed model can be used as a tool for carrying out simulations for the optimization of steel flow (in the tundish working space), in terms of achieving a high metallurgical purity of continuous casting steel.

It should be stressed that the investigation results reported in the paper constitute the first stage of a larger investigation program, which provides comprehensive research on NMI distribution in the steel flowing though the tundish. This research will be aimed at improving the hydrodynamic conditions in the tundish in terms of enhancing the liquid steel refining ability by employing flow controllers, an inert gas blow-in capability, etc.

## 2. Materials and Methods

### 2.1. Object of Research

**Figure 1:**
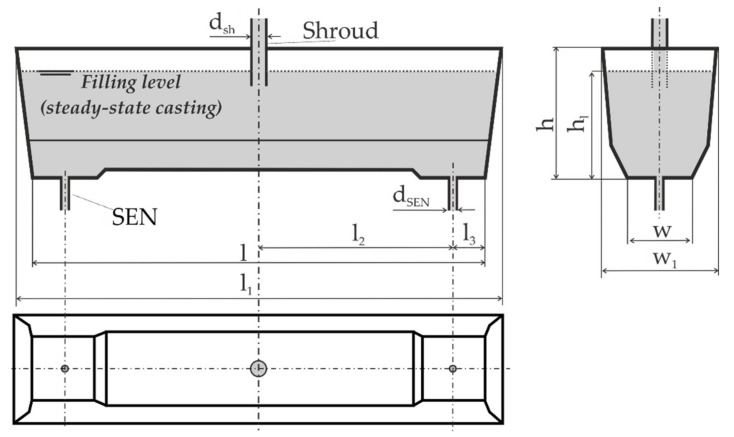
Scheme of the investigated tundish with designations of selected dimensions.

The object under study is the trough-type two-strand tundish of a Continuous Steel Casting (CSC) machine. The nominal capacity of the tundish is 60 tons of liquid steel. The tundish is installed in a plant operated in a domestic steel plant. It is used for casting (low-, medium-, and high-carbon, micro-alloy, etc.) steels smelted in the oxygen converter and subjected to ladle furnace treatment. The steel is poured into the tundish through a ceramic shield placed in its plane of symmetry. This tundish has a bottom lowered by 0.15 m in the region of outlets. The tundish is equipped with flow control devices in the normal production cycle. To preclude the influence of the flow control devices on the examined tundish phenomena, these devices were removed. The shape of the tundish together with the designations of selected dimensions is shown in Figure 1. The major dimensions of the industrial tundish and of its model are given in Table 1.

The research on the tundish was conducted on the assumption that in the conditions of the actual process, the cross-section of the semi-finished products (slab) is 1.2 × 0.2 m. The linear casting velocity of V_cast_ = 0.95 m·min^−1^ = 0.0158 m·s^−1^.

### 2.2. Models Description Used in Current CFD Calculations

Modeling of microparticle distribution in the flow reactor (the tundish model) requires solving a complex problem, which is the two-phase (liquid–solid) flow. The interaction between phases during the multiphase flow can be described mathematically based on two solving methods, namely: the Euler–Lagrange method or the Euler–Euler method.

In the case of the Euler–Lagrange method, one of the phases is regarded as a continuous phase, while the other, as a discrete phase. For the Euler–Euler method, both phases are regarded as continuous, with the respective members responsible for the interaction between them. The description according to the Euler concept, being the closest to the real two-phase flow conditions [36], is characterized by a long duration of numerical computation. The description by the Euler–Lagrange approach, in turn, as being less precise, is computationally more effective. Both methods find application in the multiphase models implemented in the ANSYS Fluent program [37]. The models characterized below were employed in the author’s study.

The Euler–Lagrange method is utilized in the Discrete Phase Model (DPM) model. In this model, the liquid is considered a continuous phase and described with the Navier–Stokes equations, whereas the solid phase is described by tracking a large number of particles passing through the liquid, while considering their influence on the continuous phase. It is applied to systems, where the volumetric fraction of the discrete phase does not exceed 10%.

The Euler–Euler method is utilized in two models, namely the Euler–Euler and the Volume of Fluid (VOF). In the Euler–Euler model, the solid phase is required to solve the continuity and the momentum equations for each of the phases. Additionally, both phases under consideration are regarded as fully interpenetrating continuous media, identified by their respective volumetric fractions.

The VOF model, on the other hand, enables solving the problem for two or several immiscible fluids, with the possibility of determining flows with a free surface. The phases of the system are treated in it as non-interpenetrating continuous media. The model requires the equations of motion to be solved for each of the phases and allows the surface tension and adhesion forces of both phases to be considered by the source members in the momentum equations.

For the DPM model, the equation describing the motion of microparticles (the discrete phase) in the water under the influence of forces acting in the system, is expressed as [37]:

(1) dupar.dt=3μwCdRepar.4ρpar.dpar.2uw−upar.+gρpar.−ρwρpar.+Fpar.

where the Reynolds number for the microparticle is defined by the relationship:

(2) Repar.≡ρwdpar.uw−upar./μw

where u_w_ is water velocity, u_par._ is microparticle velocity, ρ_w_ is water density, ρ_par._ is microparticle density, d_par._ is microparticles diameter, μ_w_ is dynamic viscosity of water, F_par._ is additional forces acting on the microparticle, and C_D_ is the dimensionless coefficient of friction developed by Morsi and Alexander [38].

In the case of the Euler–Euler model for each of the phases under consideration, the continuity and momentum equation is solved. If no source member due to mass exchange or occurring chemical reactions exists, the continuity equation for the continuous phase (water) will take on the form of [37,39]:

(3) $$\frac{\partial \alpha_{w}\rho_{w}}{\partial t}+\frac{\partial }{\partial x_{i}}\left(\alpha_{w}\rho_{w}u_{w}\right)=0$$

and for the solid phase (microparticles):

(4) $$\frac{\partial \alpha_{\mathrm{par}.}\rho_{\mathrm{par}.}}{\partial t}+\frac{\partial }{\partial x_{i}}\left(\alpha_{\mathrm{par}.}\rho_{\mathrm{par}.}u_{\mathrm{par}.}\right)=0$$

The momentum equation for the continuous (water) phase is defined as [39]:

(5) $$\frac{\partial }{\partial t}\left(\alpha_{w}\rho_{w}u_{w}\right)+\frac{\partial }{\partial x_{i}}\left(\alpha_{w}\rho_{w}u_{w}u_{w}\right)=\frac{\partial }{\partial x_{i}}\tau_{w}-\alpha_{w}\frac{\partial p}{\partial x_{i}}+\alpha_{w}\rho_{w}g+F_{w}$$

On the other hand, the momentum equation for the solid phase (microparticles) has a form similar to that of the continuous phase. An added element is the pressure gradient for the solid phase, as can be seen in Equation (5). This member is solved in compressible regions, where the volumetric phase fraction is smaller than its maximum permissible value

(6) $$\begin{array}{c} \frac{\partial }{\partial t}\left(\alpha_{\mathrm{par}.}\rho_{\mathrm{par}.}u_{\mathrm{par}.}\right)+\frac{\partial }{\partial x_{i}}\left(\alpha_{\mathrm{par}.}\rho_{\mathrm{par}.}u_{\mathrm{par}.}u_{\mathrm{par}.}\right)\\ =\frac{\partial }{\partial x_{i}}\tau_{\mathrm{par}.}-\alpha_{\mathrm{par}.}\frac{\partial p}{\partial x_{i}}-\frac{\partial p_{\mathrm{par}.}}{\partial x_{i}}+\alpha_{\mathrm{par}.}\rho_{\mathrm{par}.}g+F_{\mathrm{par}.} \end{array}$$

where α_w_ is volume fraction of water, α_par._ is volume fraction of microparticle, τ_w_ is the stress tensor for water, τ_par._ is the stress tensor for microparticle, p is the system pressure, ρ_par._ is the pressure for the solid phase (microparticles), F_par._ is denotes additional forces acting on the microparticle, and F_w_ is denotes additional forces acting on the water.

In the author’s VOF model used in computation, the continuity equation describes the functions of the variation in volumetric fraction of the phase (water, microparticles) in time and space coordinates. The continuity equation assumes the form [37]:

(7) $$\frac{\partial \alpha_{k}}{\partial t}+u_{j}\frac{\partial \alpha_{k}}{\partial x_{i}}=0$$

where α_k_ the volumetric fraction may take on one of the three states, either: α_k_ = 1—the computational cell is filled with the k-th phase (water or microparticles), or α_k_ = 0—i.e., the cell is empty, or 0 < α_k_ < 1—the cell contains the interface formed between the water and microparticles.

On the other hand, the volume of the computational cell occupied by the liquid has the following form:

(8) $$\alpha_{k\left(\mathrm{cell}\right)}={∭}_{\mathrm{cell}}\alpha_{k}\left(x,y,z\right)\mathrm{dxdydz}/{∭}_{\mathrm{cell}}\mathrm{dxdydz}$$

### 2.3. Numerical Modeling

The CFD computations were carried out for the geometry of the tundish model. To make direct verification with the laboratory test results, water was used as a model liquid.

The working space of the facility was mapped out in the DirectModeler preprocessor [40]. The simulation using the LES model required the whole facility’s space to be mapped out. For the solution using the RANS (Reynolds–Averaged Navier–Stokes) model [37], the plane of symmetry passing through the inlet to the tundish was taken into consideration. For the geometry of the tundish model, a computational mesh was generated, which was densified in the inlet and the outlet regions. The quality of the generated computational meshes was verified using the criterial skewness angle [37]. To determine the effect of mesh size on the numerical solution using the LES (Large Eddy Simulation) method, Pope’s criterion was used [41].

The numerical simulations were made in the ANSYS Fluent program, ver. 16 [37]. A detailed description of the numerical models, along with appropriately formulated boundary conditions and computation results, is provided in work [42].

For computation using two-phase models, additional boundary conditions were specified, which are defined on the characteristic surfaces of the facility under examination. These conditions correspond to the specificity of laboratory tests. In the plane corresponding to the tundish inlet, the mass flow boundary was assumed. In its case, a given mass flux of microparticles of a specified diameter is established. The numerical analysis covered four microparticles of a size of about 20, 50, 100, and 140 μm, respectively.

In the simulation it was assumed that microparticles enters the tundish with constant initial velocity, equal to water velocity. At the inlet (shroud) a “mass flow inlet” boundary condition was used for microparticles. On the walls (side walls and bottom of the tundish) a “reflect” boundary condition was used for microparticles, whereas microparticles exit the domain through the top free surface (“escape” boundary condition), which corresponds to ideal conditions for absorption. Microparticles also can exit the domain through the outlet surface where the “escape” boundary condition has been used.

**Figure 2:**
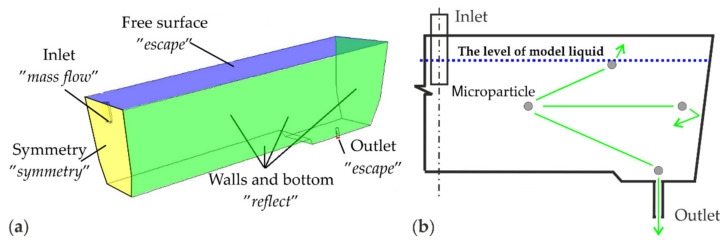
The boundary conditions for the developed multiphase models: (**a**) computational domain assigned to the planes, (**b**) diagrammatic view.

A graphical representation of all boundary conditions for the multiphase models is shown in Figure 2.

P-V coupling was resolved using the SIMPLE scheme. Pressure equation was resolved using the PRESTO or Second Order method. For discretization of other equations, the authors used the second order upwind scheme. To determine whether the solution is converged, the residuals of variables were checked at each time step to make sure they were smaller than 1 × 10^−6^.

The designations of the computational variants and the description of the parameters used in CFD computation are shown in Table 2.

### 2.4. Physical Modeling

The laboratory measurements were carried out with the use of the physical CSC machine model. The model is characterized by its segmental structure. Individual constructional parts of the model belong to the main segments and the auxiliary segments. The model approach, based on such a structure (segmental), ensures that the assumed functionalities of the test stand are obtained. The basis of the entire model is a structural element, which in this case is a tundish made of plexiglass (reduced scale). It is there that experimental measurements of the phenomena, that are the subject of the research, were carried out. The relevant similarity criteria [43], required to be met for the scaled down models, needed to be kept for this segment. The described model had already been used to conduct earlier research [25,42].

Obtained test results from the water model could be moved to real objects as long as similarity criteria were correctly applied. In order to do so, both geometric and dynamic similarities [42,43] should be satisfied.

It is very hard to reach the total similarity of flows. One of the examples could be an incapability to satisfy Froude and Reynolds critical numbers simultaneously in models by reducing or increasing the linear scale. Both criterion numbers can only be satisfied at the same time when models are made on linear scale of S_L_ = 1. Therefore, partial similarity is commonly regarded as sufficient. As a result, either one or two important quantities in the investigated flow are selected by determining the so-called dominating criterion [5,7,42,43]. In the authors’ study, the dominant criterion was the Froude number:

(9) $${Fr}^{\prime }=\frac{{u^{\prime }}^{2}}{g^{\prime }\times L^{\prime }}=\frac{u^{2}}{g\times L}=\mathrm{Fr}$$

where L′ is the dimension characteristic of the model, L is the dimension characteristic of the real object, u′ is the fluid velocity of the model, u is the fluid velocity, and g is the acceleration due to gravity.

Based on the Fr criterion, the kinematic similarity of the model to the industrial conditions was determined. The scale method was employed to determine the scale of the model liquid flow intensity, S_Q_, according to Equation (10):

(10) $$S_{Q}=\frac{S_{L}^{3}}{S_{t}}=\frac{S_{L}^{3}}{S_{L}^{1/2}}=S_{L}^{5/2}$$

where S_Q_ is the volume flow scale, S_L_ is the linear scale of the model, and S_t_ is the time scale.

For describing the motion problem (the distribution of nonmetallic inclusions) in liquid steel flow in the tundish, it is insufficient to only determine the dynamic and kinematic similarities of model fluid flow. One should also consider the interaction between nonmetallic inclusions and the liquid steel, but this is difficult to use to resolve such a complex problem in an explicit manner. It is expected that the buoyant forces and the particle motion dynamic in the model liquid will be similar to the behavior of inclusions in the industrial tundish. Determining similarity for the motion of nonmetallic inclusions in the tundish and motion of microparticles in the investigated tundish water model generates problems. An issue is defining proper conditions, which ensures proportionality between fluid flow and particle flotation velocities [25,27]. Therefore, the selection of microparticles for testing using the physical model must be made while following specific rules. The material from which they are constructed and their physical form may be different. However, they must meet specific physicochemical conditions. These conditions are defined based on the dimensional analysis of the mathematical model of interaction between the microparticles and the model liquid and the determination of the required similarity conditions. Mathematical procedures for such computations are given in references [25,27]. It should be remembered that during the analysis of the transfer of testing results to the real conditions, appropriate conversions must be made.

For the implementation of the presented model tests, the distribution of non-metallic inclusions in the liquid steel flowing through the tundish, hollow glass microspheres (Scotch 3M, type K1) were used [44]. They make it possible to carry out the planned research because they reflect the laser light. Moreover, they map the actual inclusions, which are characterized by an aspect ratio close to one and meet the similarity criteria, because the ratio of microparticle density to water specific density is 0.12. It was therefore assumed that these particles could be used to model the behavior of non-metallic inclusions in liquid steel.

The model studies of the microparticle distribution process were divided into two categories, namely: the qualitative analysis (using the visualization techniques) and quantitative analysis (using the laser techniques to count particles) [45].

**Figure 3:**
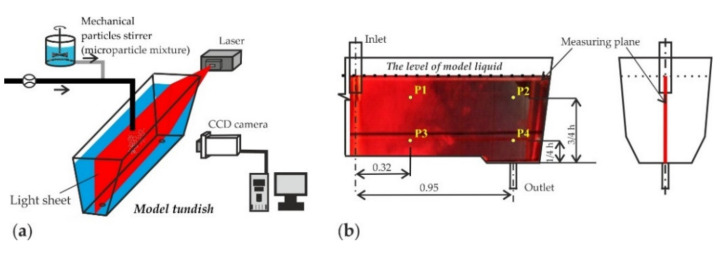
(**a**) Schematic view of the performed experiment, (**b**) Picture showing the location of characteristic points for measurement in the investigated object. Reproduced with permission from ref. [25]. Copyright 2019 WILEY-VCH Verlag GmbH & Co. KGaA, Weinheim.

In order to obtain images (visualizations) of microparticles moving in the water, it was necessary to illuminate the tundish model with a laser beam directed for specific planes (Figure 3a). The obtained pictorial material enabled the recording (through the photographs) of microparticle distribution in the reactor at specific time intervals.

The quantitative analysis was carried out by measuring the number of solid particles in a given volume of liquid taken during the experiments. Then, the samples were tested with the use of a Klotz particle counter—Abakus mobil fluid [45]. The selection of the sampling locations was crucial for a representative assessment of the actual distribution of the microparticles in the liquid. The location of the measurement points was determined on the basis of the results of preliminary numerical simulations of the marker scattering in the liquid. It was decided that the monitors would be placed on the longitudinal plane of the tundish, as for measurements with the visualization techniques (Figure 3a), passing through the centers of inlet and outlets. The location of the points is shown in Figure 3b. Measurements were done by monitoring the number of particles flowing at specific time intervals (quantitative analysis). Samples taken from specific points of the tundish model (P1, P2, P3, P4—Figure 3b) were tested with the use of a particle counter.

The methodology of the laboratory examination of the microparticle distribution process is described in detail in work [25]. The basic physical quantities considered in the laboratory tests are shown in Table 3.

## 3. Results and Discussion

The characteristics of the forecast state of liquid motion for the water–microparticle mixture provide significant information on the condition of liquid flow through the tundish under study.

However, this motion pattern should not be transferred to either all or individual elements of the liquid. The liquid contains solid particles in different sizes, whose trajectories may not necessarily coincide with the senses of the liquid vectors. It should also be remembered that buoyant forces resulting from differences in specific density act upon the microparticles.

An adequate analysis of the motion and distribution of microparticles in the liquid as it flows through the tundish model is provided by the analysis of the contour maps of microparticle concentrations.

### 3.1. Results and Validation of Microparticles Distribution in the Tundish Model

**Figure 4:**
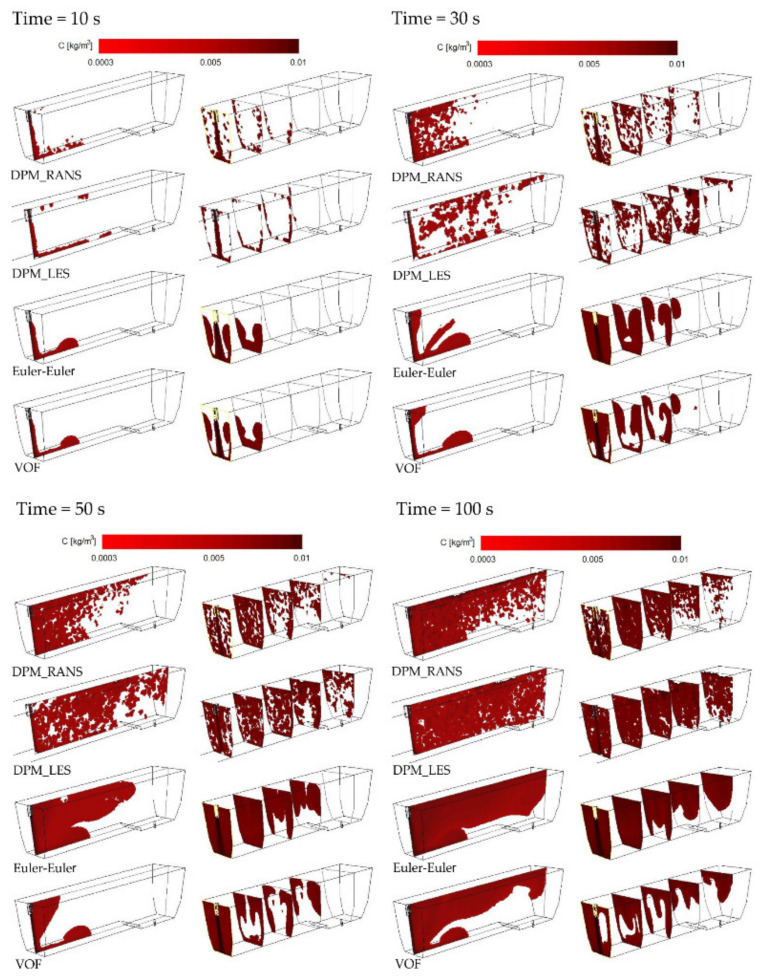
Contour maps of microparticles distribution (20 μm) on the control planes.

**Figure 5:**
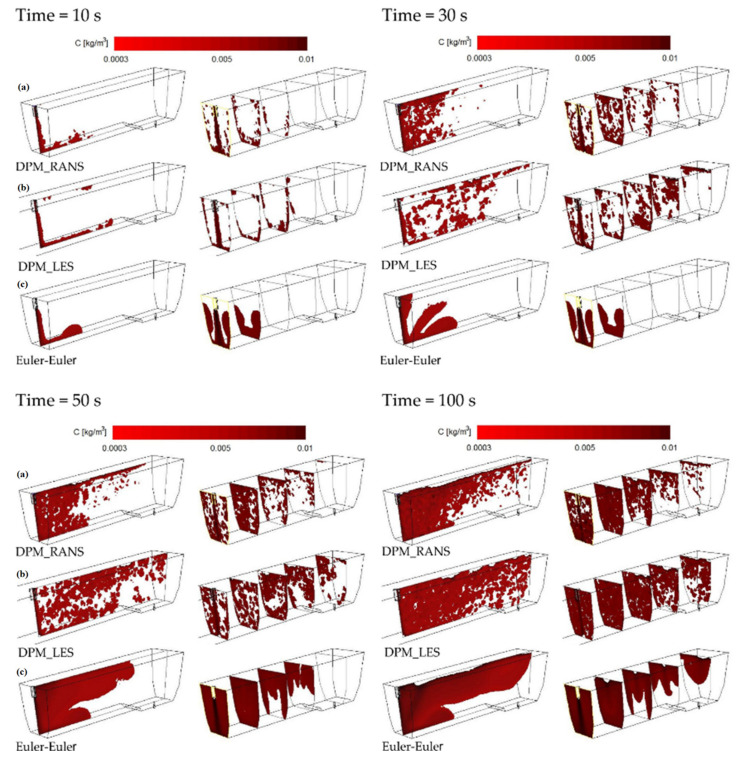
Contour maps of microparticles distribution (50 μm) on the control planes: (**a**) DPM_RANS; (**b**) DPM_LES; (**c**) Euler–Euler.

**Figure 6:**
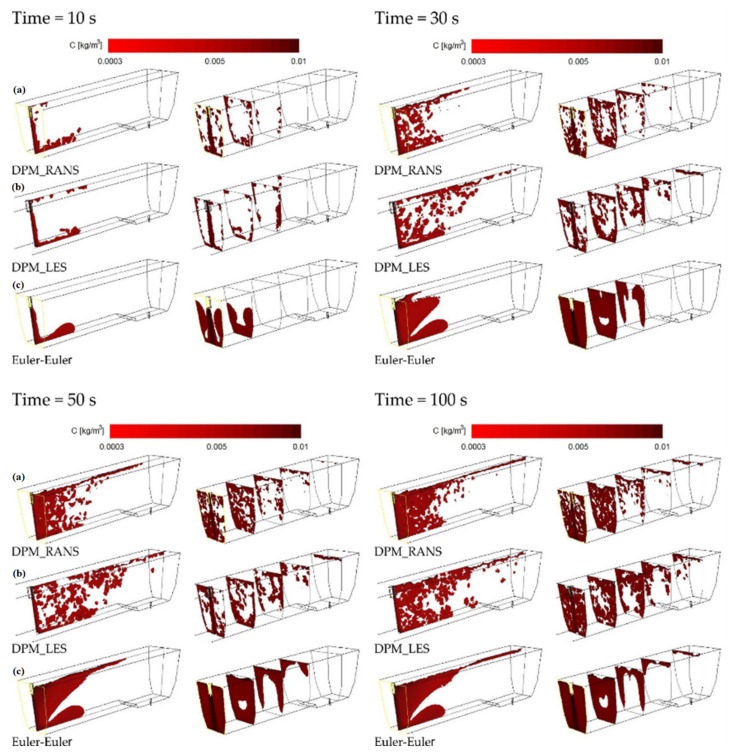
Contour maps of microparticles distribution (100 μm) on the control planes: (**a**) DPM_RANS; (**b**) DPM_LES; (**c**) Euler–Euler.

The CFD computation results representing the motion and distribution of microparticles are shown in the defined control planes (the vertical longitudinal plane—coming through the outlet and the inlet (y = 0 m) and the five vertical transverse planes (x = 0 m, x = 0.2 m, x = 0.4 m, x = 0.6 m, and x = 0.855 m). The numerical computation covered four microparticle sizes. Sample patterns of the contour maps of computed microparticle concentrations for four computation variants are shown in Figure 4, Figure 5 and Figure 6.

The analysis of the contour maps represents the distribution of microparticles of a size of about size 20 μm (Figure 4) and shows that the pattern of flow (motion) of microparticles for VOF models differs from the remaining models (computation variants). Moreover, for the VOF model, problems with obtaining the solution convergence were encountered, therefore a decision was made to abandon simulations using this model.

When comparing the contour maps for the distribution of microparticles of a size of 50 μm (Figure 5) for durations of 50 and 100 s, a similar microparticle flow pattern was observed for the DPM_RANS (Figure 5a) and the Euler–Euler models (Figure 5c). The flow pattern for the DPM_LES (Figure 5b) model, on the other hand, looks totally different. The apparent difference is a tendency to more quickly flow up to the water free surface and a considerably greater dissipation of microparticles within the tundish space.

The analysis of the results illustrated in Figure 6 (the distribution of microparticles of a size of 100 μm) shows also that the DPM_RANS (Figure 6a) and the Euler–Euler (Figure 6c) models describe the pattern of flow (motion) of microparticles in the model liquid flowing through the tundish in a comparable manner.

**Figure 7:**
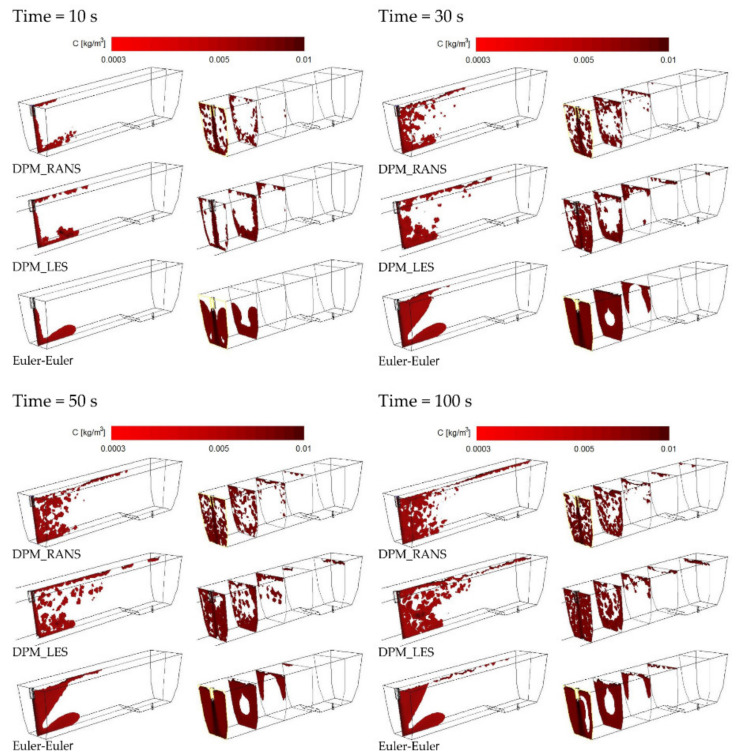
Contour maps of microparticles distribution (140 μm) on the control planes.

Upon the analysis of the microparticles distribution results for all considered sizes (Figure 4, Figure 5, Figure 6 and Figure 7) in the aspect of microparticles motion and distribution, it is evident that at a time of t = 10 s, the microparticle flow pattern is very similar for all sizes and models considered. With time, depending on the size of microparticles and the models used, a different flow pattern is noted. However, one common feature is apparent on the longitudinal section of the tundish, which is the dissipation of microparticles within the inlet zone and, analogous to all considered microparticle sizes, a tendency to flowing at the bottom (up to approximately 0.22 m from the inlet). This was confirmed by the analysis of the transverse planes (the first control plane x = 0.2 m). By contrast, at a farther distance from the inlet, the distribution of flowing microparticles is different and already substantially depends on the microparticle size. Particles with larger diameters travel towards the free surface, while particles with smaller diameters accumulate in the lower regions of the model. Microparticles of a size of up to 20 μm the fastest reach the tundish outlet, flowing through the entire available working space of the examined tundish. Microparticles of a size of up to 50 μm, similarly, flow through the available space, but reach the outlet much slowly. By contrast, microparticles of a size of up to 100 and 140 μm (Figure 6 and Figure 7) show a tendency to flowing up to the water table surface (from 0.4 m from the inlet—the control plane x = 0.4 m), which indicates a growing predominance of buoyant forces in the local field of forces.

The correctness of the mathematical models (computation variants) used in the CFD computations was verified by comparing them with laboratory measurement results. The validation was done for the DPM_RANS, DPM_LES and Euler–Euler computation variants. Four microparticle grain-size fractions were selected for comparative analysis, namely: 20, 50, 100, and 140 μm. For this purpose, the results describing the motion and distribution of microparticles in the liquid flowing though the tundish were used. The comparison was made in two aspects: qualitative and quantitative. In the qualitative comparison, the results of the determination (motion visualization) of the motion and spatial distribution [25] of microparticles, derived from empirical tests, were juxtaposed with the results obtained from the CFD simulations. For the quantitative validation, microparticle distribution curves were used.

**Figure 8:**
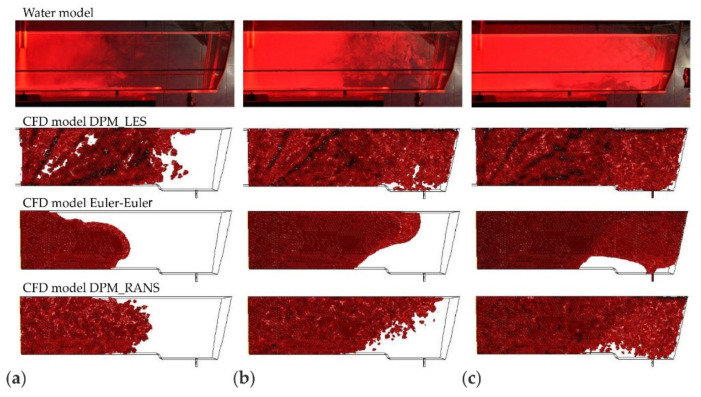
The location of the microparticles (fraction 20 μm) in the tundish model for a duration of, respectively: (**a**) 25, (**b**) 60, (**c**) 120 s.

**Figure 9:**
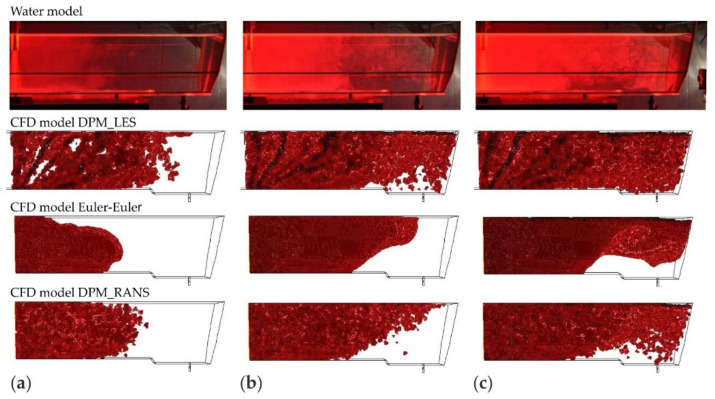
The location of the microparticles (fraction 50 μm) in the tundish model for a duration of, respectively: (**a**) 25, (**b**) 60, (**c**) 120 s.

**Figure 10:**
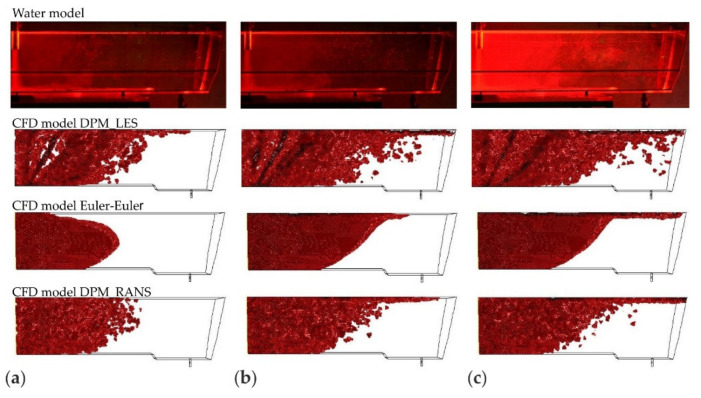
The location of the microparticles (fraction 100 μm) in the tundish model for a duration of, respectively: (**a**) 25, (**b**) 60, (**c**) 120 s.

**Figure 11:**
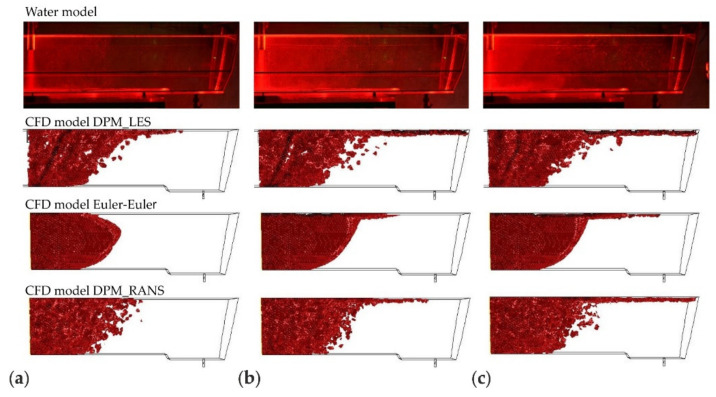
The location of the microparticles (fraction 140 μm) in the tundish model for a duration of, respectively: (**a**) 25, (**b**) 60, (**c**) 120 s.

The juxtaposition of the results of microparticle motion and distribution is shown in Figure 8, Figure 9, Figure 10 and Figure 11. The presented results are systematized in the following order: first, the laboratory measurements taken on the water model. Second, the CFD computations, respectively, for the computation variants—models DPM_LES, Euler–Euler, and DPM_RANS. To objectivize this analysis, three durations (25, 60, and 120 s) were chosen, at which time determinations were made.

By analyzing the motion of microparticles in the liquid flowing through the tundish model, shown in Figure 8, Figure 9, Figure 10 and Figure 11, from the point of view of the consistency of the mathematical models used in CFD computations, very high consistency between the experimental measurement results and the results of computations made using the DPM_RANS model can be shown. This consistency is observed for all microparticle sizes examined, for the microparticle flow front and motion trajectory.

For the results of simulations using the Euler–Euler model, similarity with the experimental results is also noticed to a great extent. However, some differences occur, which are visible after a duration of 25 s for all analyzed microparticle fractions (cases (a) in Figure 9, Figure 10 and Figure 11). These show up in the microparticle motion picture (a different microparticle flow front).

On the other hand, the comparison of the experimental (laboratory) measurement results with the computation results for the model DPM_LES model look completely different. The CFD computation results deviate considerably from the laboratory determination results. A faster movement of the front of flowing microparticles is observed, and significant differences in the motion trajectory of microparticles, depending on their size, is noted.

The comparison presented in (Figure 8, Figure 9, Figure 10 and Figure 11) clearly shows that the DPM_RANS and Euler–Euler models similarly describe the motion of microparticles in the volume of the model liquid flowing through the tundish. Nevertheless, the microparticle distribution obtained from CFD computations using the DPM_RANS model is the most consistent with the empirical distribution. A similar microparticle motion pattern and, equally importantly, a similar outlet reaching time are visible.

Proceeding with quantitative validation, the CFD simulation results—including the mass concentration of microparticles—were compared to analogous experimental data derived from model studies done at the measurement points (P1, P2, P3, and P4) under examination. The position of points in the tundish space for CFD computation corresponds strictly to the position of the measurement points from the empirical studies. Because of the discrete nature of the measurement points for the RANS and LES models, a SAE J211/1 numerical filter was employed for obtaining the required results.

**Figure 12:**
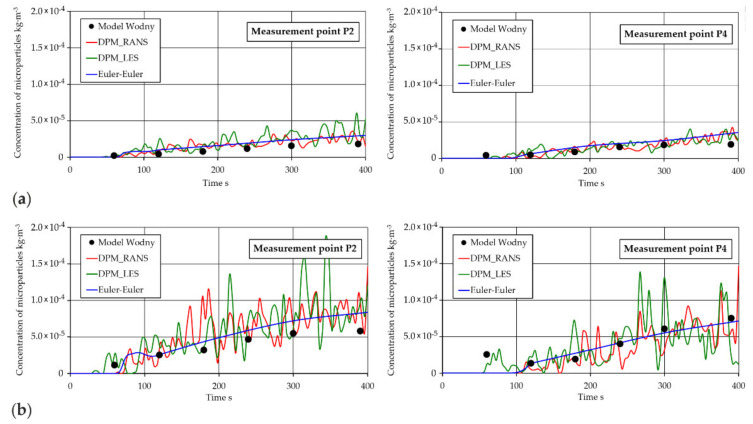
Concentration of microparticles at measuring points: (**a**) 20 μm, (**b**) 50 μm.

A sample summary of experimental determination results and CFD results for microparticle concentrations at the measurement points is shown in Figure 12. The summary concerns the measurement points located in the outlet zone. The concentration of microparticles in this zone represents the state after the microparticles have gone through the entire tundish volume. From the point of view of industrial practice, such a solution ensures the most objective determination of the metallurgical purity of liquid steel flowing into the mold.

The results represented in Figure 12 enable direct comparison of experimental measuring results with CFD simulation results. They provide information about motion and distribution in the form of variations in microparticle concentration in the tundish working space at different measurement times. Satisfactory consistency was noticed for all models used in computations compared to the microparticle concentration values obtained at the measurement points.

Less similarity between the empirical results and the numerical simulation results was noted with the increase in the size of microparticles flowing in the liquid. This is due to considerably greater buoyant forces characteristic of those microparticles, as a result of which, at an early motion stage, they were already carried up to the free liquid surface and then stayed there.

To sum up, the detailed qualitative and quantitative analysis of the obtained microparticle distributions and concentration values shows that the DMP_RANS guarantees the best consistency with the empirical determination results.

### 3.2. Discussion

The flow of liquid steel in the tundish, which at the same time illustrates that the distribution of nonmetallic inclusions is a complex hydrodynamic problem. Therefore, it is crucial to learn and understand it. The knowledge acquired by means of theoretical analyses of phenomena under consideration would often be insufficient. It is much more important to obtain verified information experimentally. Direct studies of such hydrodynamic phenomena under industrial conditions are very difficult and costly. Methods that enable obtaining results similar to those obtained in industrial conditions are numerical simulations carried out using specialized CFD software and laboratory tests done on water models. By juxtaposing both research methods, a numerical model has been selected, which best reproduces the phenomenon of NMI distribution in the tundish under study.

The analysis of the presented results of the numerical simulation and physical experiment allows to state that the microparticle distribution process is better described by the Euler–Lagrange method. The remaining models relying on the Euler–Euler description method exhibit greater discrepancies in the results and—in the case of the VOF model—problems with obtaining the convergence of the solution. It can therefore be inferred that the Euler–Euler method, theoretically closer to two-phase flow conditions, not always correctly renders the distribution of solid microparticles in the liquid. Comparison of characteristics measured in the water model—the distributions and local concentrations of microparticles—with respective characteristics determined in the DPM model has shown that the results obtained using this model reproduce the empirical distribution with great accuracy. A similar pattern of the motion of microparticles and similar their time flow characteristics were recorded. Comparative analysis of the laboratory measurements with the numerical simulation results has demonstrated that the discrete phase model (DPM) with formulated initial and boundary conditions correctly describes the motion of solid particles in the flow under examination. The distribution of microparticles depends on the liquid flow velocity, thus being different for individual flow zones in the tundish. At high velocities of the liquid (the inlet region–the turbulent flow zone), microparticles are carried away with the liquid stream. At lower velocities (the remaining tundish region–the laminar flow zone), the distribution of microparticles is determined more by buoyant forces, this being especially true for large-diameter microparticles. The distribution of microparticles in the liquid flowing through the tundish under study is closely dependent on their sizes. The majority of microparticles of a size of 20 μm move at the tundish bottom. Microparticles of a size of 50 μm flow considerably slower and essentially fill the whole available working space of the tundish. By contrast, large microparticles (of a size of 100 and 140 μm) are characterized by fast flowing up to the free surface. The distribution of microparticles also depends on the tundish region (the inlet region or the remaining tundish region) in which a given microparticle is situated. This refers to the velocity of liquid flow in the tundish and its related turbulence. At high liquid velocities (the inlet region—the turbulent flow zone), microparticles are carried away with the liquid stream. At lower velocities (the remaining tundish region—the laminar flow zone), the microparticle distribution is determined by buoyant forces, which is especially evident for large-diameter microparticles. The flow at the inlet of the tundish is turbulent, which causes flotation of particles, especially those with small diameters (20 μm), and their lifting towards the free surface. In other regions characterized by laminar flow, the microparticles fall down, therefore the flow pattern in the tested tundish should be modified.

## 4. Conclusions

The article presents the results of a critical analysis of the effectiveness of using some CFD solutions to determine the motion of non-metallic inclusions in liquid steel. Numerical simulations were verified in experiments carried out on the water model. The obtained results enabled the formulation of the following conclusions:

- the process of microparticle distribution is best described by the Euler–Lagrange method,
- distribution characteristics and local concentrations of microparticles, determined on the basis of the DPM model, reflect the empirical distribution with a high accuracy,
- the distribution of microparticles depends on the velocity of the liquid flow and thus differs for the different flow zones in the tundish,
- microparticles of a size of 20 µm generally move at the tundish bottom,
- microparticles of a size of 50 µm flow considerably slower and essentially fill the whole available working space of the tundish,
- large microparticles (with a size of 100 and 140 μm) are characterized by fast flowing up to the free surface,
- turbulent flow in the infusion zone promotes the discharge of microparticles, especially of small sizes (20 µm).
- The investigation results reported in this paper constitute the first stage of a larger research program, which envisages comprehensive studies of the distribution of nonmetallic inclusions in steel flowing though the tundish. The developed and improved model will include a modified boundary condition at the liquid-gas interface (liquid free surface), which describes whether a particle is reflected or absorbed by the surface, depending on the critical velocity of each particle that reaches the free surface. This condition will be introduced with the user-defined function UDF. We will as also modify a tool for carrying out tests for the optimization of flow in the tundish working space using flow control devices. These tests will aim to improve the hydrodynamic conditions in tundishes in terms of enhancing their liquid steel refining capabilities. The effect of this will be the obtaining of high metallurgical purity of continuously casted steel.

## Figures and Tables

**Table 1:** Design data for the industrial and the model tundish.

Parameter/Unit	Symbol	Tundish	
		Industrial	Model Scale 1:4
Volume, m^3^	V	8.550	0.130
Height (filling level), m	h_l_	1.200	0.300
Height, m	h	1.460	0.365
Length, m	l	7.600	1.900
	l_1_	8.160	2.040
Width, m	w	0.720	0.180
	w_1_	1.260	0.315
Outlet (SEN) position location, m	l_2_	3.800	0.950
	l_3_	0.400	0.100
Inlet (shroud) diameter, m	d_sh_	0.052	0.013
Outlets (SENs) diameter, m	d_SEN_	0.090	0.023

**Table 2:** Description of parameters used for various simulation models.

Parameter	CFD Simulation Variants			
	DPM_RANS	DPM_LES	Euler–Euler	VOF
Investigated object	half of the object	entire facility	half of the object	half of the object
Mesh Size	282,000	550,400	282,000	282,000
Mesh Quality criterion	Q_EAS_ = 0.5	M_p_ = 0.175	Q_EAS_ = 0.5	Q_EAS_ = 0.5
Turbulence method	RANS	LES	RANS	RANS
Multiphase flow method	Euler–Lagrange	Euler–Lagrange	Euler–Euler	Euler–Euler
Thermal boundary condition	Isothermal			
Volume flow rate (water), m^3^·s^−1^	1.29 × 10^−4^	2.58 × 10^−4^	1.29 × 10^−4^	1.29 × 10^−4^
Water density, kg·m^−3^	998.2			
Water kinetic viscosity, kg·m^2^·s^−1^	1 × 10^−6^			
Microparticles density, kg·m^−3^	120			
Microparticles diameter, μm	20, 40, 100, 140			
Mass flow (microparticles), kg·s^−1^	6.25 × 10^−^^7^	1.25 × 10^−^^6^	6.25 × 10^−^^7^	6.25 × 10^−^^7^
Time step, s	0.1	0.01	0.1	0.1
Simulation time, s	400			

**Table 3:** Physical quantities—laboratory research.

Parameter	Symbol	Unit	Value
Volume flow rate	Q_v_	m^3^·s^−1^	2.58 × 10^−4^
Water density	ρ_w_	kg·m^−3^	998.2
Froude number	Fr	-	2.10 × 10^−5^
Microparticles density	ρ_par._	kg·m^−3^	120
Microparticles diameter	d_par._	μm	10 ÷ 140
Ratio density of microparticle to water density	-	-	0.1202

## Data Availability

Data is contained within the article.
